# Withdrawal pain following patients discontinuing Trk inhibitors

**DOI:** 10.1177/10781552241279196

**Published:** 2024-08-27

**Authors:** Alan Chin, Sheila Lindsay, Emily K Bergsland, Hyunseok Kang

**Affiliations:** 1 Department of Pharmacy, 8785 University of California San Francisco Medical Center, San Francisco, CA, USA; 2 8785 University of California, San Francisco-Helen Diller Family Comprehensive Cancer Center, San Francisco, CA, USA; 3 Department of Medicine, Division of Medical Oncology, 8785 University of California, San Francisco, CA, USA

**Keywords:** NTRK, Trk inhibitors, larotrectinib, entrectinib, withdrawal pain

## Abstract

**Objective:**

This article aims to expand on the existing literature regarding the incidence of withdrawal pain following discontinuation of Trk inhibitors and to explore strategies that mitigate this withdrawal pain.

**Data Source:**

A retrospective observational study was conducted among patients who were at least 18 years-old or older and had documentation of starting larotrectinib or entrectinib at University of California, San Francisco (UCSF) between November 2018 and November 2022. Data were collected from electronic records and pharmacy databases and a total of 21 patients were identified in this study.

**Data Summary:**

Of the 21 patients included in this study, five patients (24%) experienced pain during temporary or permanent discontinuation of Trk inhibitor with the onset of withdrawal pain ranging from a few hours to three days following discontinuation. Various strategies were implemented to manage this pain including restarting of Trk inhibitor, tapering of Trk inhibitor on discontinuation, minimizing dose interruptions and use of prescription pain medications.

**Conclusion:**

This article illustrates the presence of withdrawal pain syndrome in patients stopping a Trk inhibitor treatment and highlight the need for patient education to avoid missing any doses and for development of a guideline for Trk inhibitor discontinuation.

## Introduction

Neurotrophic tyrosine receptor kinase *(NTR*K) genes encode tropomyosin receptor kinase (Trk) proteins that help regulate pain, proprioception, appetite, and memory.^1,2^ Chromosomal rearrangements of *NTRK* genes are found across diverse tumor types that occur in children and adults leading to Trk fusion proteins in less than 1% of all solid tumors.^2,3^ Larotrectinib and Entrectinib are both highly selective Trk inhibitors that are FDA approved for the treatment of *NTRK* gene fusion positive cancers across all solid tumor types. Repotrectinib, another highly selective Trk inhibitor, has also recently obtained FDA approval for the same indication.

Trk proteins are typically expressed in the nervous system and play an important part of neuronal development and differentiation as evidenced by the development of congenital insensitivity to pain (CIPA) hereditary disorders in patients with *NTRK1* gene mutations leading to loss of function TrkA.^2^ Based on the *NTRK1* gene's role in pain sensation, ligands for TrkA, such as nerve growth factor (NGF), and TrkA have been a target for pain analgesics with NGF function reported to induce pain in adults.^4–6^ Patients who discontinued Trk inhibitors experience varying symptoms of withdrawal pain described as full-body aches, muscle pain, allodynia, and flares of pre-existing pain symptoms and can occur in approximately 34% of patients.^7,8^ Few studies comment on this withdrawal pain and mechanisms to describe this withdrawal pain remain to be elucidated^.^^7^ This article seeks to expand on the available literature regarding the incidence of withdrawal pain following discontinuation of Trk inhibitors. Specifically, we are interested in exploring management strategies that decrease the risk of withdrawal pain following Trk inhibitor discontinuation. Here, we report a case of a patient successfully titrated off from larotrectinib following disease progression despite significant body aches/pains after missing two days of larotrectinib. We additionally report data from our institution which suggest a correlation between Trk inhibitor discontinuation and withdrawal pain onset.

## Case report

A 47 year-old female patient was initially diagnosed with a metastatic grade 3 neuroendocrine tumor likely arising from small bowel with extensive liver metastases with bulky liver disease (Ki-67 25.4%) and bone involvement. She subsequently received palliative systemic therapy with 3 cycles of leucovorin, fluorouracil and oxaliplatin (FOLFOX) along with octreotide, a somatostatin analogue, but only had minimal benefit with slight growth of the tumor. A comprehensive genomic profiling using a capture-based next generation sequencing (UCSF500)^9^ identified a complex rearrangement involving NTRK3, ATF7, and TRPM8 with the translocation junction located within intron 13–14 of the NTRK3 gene and the downstream exons of NTRK3 encoding the intracellular tyrosine kinase. The molecular tumor board recommended a highly specific Trk inhibitor based on the high likelihood this was a true fusion involving *NTRK3* that was further supported by the high number of reads detected over the translocation breakpoint. Patient started larotrectinib 100 mg twice daily while continuing octreotide and tolerated the regimen well with mild adverse events such as generalized fatigue, weight gain, and increase in appetite. She remained on larotrectinib for 17 months until follow up Dotate-PET imaging showed a mixed response with more osseus metastases and stable liver metastases with clinically more carcinoid related symptoms necessitating a treatment change. During this time, she also noted change in symptoms such as leg cramps at night, increased fatigue, and occasional generalized body aches/pains that were aggravated if she misses more than 2 days of larotrectinib. The decision was made to taper her off from Larotrectinib by offering Larotrectinib 100 mg once daily for 1 week and then stopping to minimize the risk of withdrawal pain or pain exacerbation. Patient tolerated this titration well with no complaints of body aches/pains. Informed consent was obtained from the patient for publication.

## Results

We retrospectively reviewed the institutional data at University of California, San Francisco (UCSF) between November 2018 and November 2022. Approval for this study, including a waiver of informed consent and a Health Insurance Portability and Accountability Act waiver of authorization, was obtained from the Institutional Review Board (IRB # 22-36854). Patients who were at least 18 years-old or older and had documentation of starting larotrectinib or entrectinib were deemed to be eligible. Pain was graded according to Common Terminology Criteria for Adverse Events (CTCAE) version 5.0.^10^ Demographics and withdrawal pain management were obtained from electronic records and pharmacy databases.

Total of 21 patients were included in the data analysis. Of these 21 patients, majority had non-small cell lung cancer (NSCLC) or thyroid cancer (Table 1). Fifteen patients (71%) had *NTRK* gene rearrangements and 6 patients (29%) harbored a *ROS1* gene fusion as entrectinib has efficacy in *ROS1* fusion positive NSCLC. Thirteen patients (62%) had received larotrectinib and 8 patients (38%) had received entrectinib including 6 patients with *ROS1* fusion. Fourteen patients (67%) remained on therapy with either larotrectinib or entrectinib at the time of last visit. Five patients (24%) experienced pain during temporary or permanent discontinuation of Trk inhibitor with the onset of withdrawal pain ranging from a few hours (2/5; 40%) to 3 days (1/5; 20%) following discontinuation. The remaining 2 patients did not have this information documented. Of these 5 patients, 60% of them noted grade 1 pain and 40% of them noted grade 2 pain and experienced immediate pain relief with restart of Trk inhibitor within 30 min to a few hours. Three out of 5 patients (60%) stated that not missing doses was an effective strategy in avoiding withdrawal pain and only one patient (20%) was titrated off from the Trk inhibitor to avoid withdrawal pain when the patient was taken off from the treatment for disease progression. None of the patients required hospitalization for management of withdrawal pain. Specific symptoms associated with the withdrawal ranged from muscle aches/weakness to leg/knee joint pain and described as muscle cramping that worsens with movement. One patient also noted toothache as well. They were managed with either restart of Trk inhibitor, taper of Trk inhibitor on discontinuation, minimizing dose interruptions and/or prescription pain medications such as tramadol. One patient noted that pain resolved 2 days after discontinuation of larotrectinib. Non-steroidal anti-inflmmatory rugs and acetaminophen were tried in 2 patients but were noted to be ineffective. However, tramadol was noted to lead to partial pain relief in 1 patient. The most effective management strategy appeared to be either restarting or titrating off of Trk inhibitor.

**Table 1.** Demographic and clinical characteristics of the patients.

Characteristics	Patients N = 21 (100%)
Sex	
Male	11/21 (52%)
Female	10/21 (48%)
Age	42–89 years old
Diagnosis	
Non-Small Cell Lung Cancer	9/21 (43%)
Thyroid Cancer	4/21 (19%)
Pancreatic Cancer	2/21 (10%)
Ampullary Cancer	1/21 (5%)
Anaplastic Astrocytoma	1/21 (5%)
Breast Cancer	1/21 (5%)
Neuroendocrine Tumor	1/21 (5%)
Uterine Leiomycosarcoma	1/21 (5%)
Unknown Primary (likely mucus producing adenocarcinoma)	1/21 (5%)
Genomic Alterations	
*NTRK1/2/3* fusion/rearrangements	15/21 (71%)
*ROS1* fusion	6/21 (29%)
Prescribed Trk Inhibitor	
Larotrectinib	13/21 (62%)
Entrectinib	8/21 (38%)

## Discussion

*NTRK1/2/3* genes encode TrkA/B/C proteins, respectively, and help regulate pain, proprioception, appetite, and memory.^1,2^ Chromosomal rearrangements leading to *NTRK* fusion genes then result in constitutive activation of Trk proteins which then act as oncogenic drivers of tumor growth.^3,4^ *NTRK* gene fusions are a relatively rare occurrence with a prevalence of 0.3% across 45 cancers with the highest prevalence observed in salivary gland tumors (2.28%).^3^

Much remains to be elucidated in terms of understanding the mechanisms of Trk inhibitors and how the TRK pathways regulate the nervous system. NGF has been associated with pain modulation and increased NGF signaling has been shown to produce pain and hyperalgesia.^4^ Pain flares can be debilitating with immense impacts on a patient's quality of life. Pain flares with Trk inhibitor discontinuation likely stems from the sudden interruption of these inhibitory effects on nerve cell signaling and could be related to the effect of NGF on pain modulation.^7^ Few studies comment on this withdrawal pain phenomenon and only one study to date has identified possible management strategies for mitigating this withdrawal pain with a small sample size.^7^ Taper of Trk inhibitors has been reported as a formidable strategy in mitigating withdrawal pain when discontinuing treatment but only two patients described in the previously mentioned study were tapered off in differing strategies without a clear consensus.^7^ Another article suggests that interruptions in Trk inhibitors should be avoided and to consider a slow taper for patients who discontinue treatment but provide minimal evidence to support this recommendation.^8^ This study adds to the current literature and highlights an effective strategy to mitigate withdrawal pain upon Trk inhibitor discontinuation. Cost and tolerability are two aspects that should be considered when seeking this titration approach as there could be the risk of financial toxicity such that the patient can no longer continue to afford taking this high-cost medication for an additional duration of time or that the patient can no longer tolerate the medication side effect profile and must weigh the risk and benefits of immediately stopping this drug without weaning off.

Similar to the data presented by Liu et al.,^7^ our data is also sparse with 21 patients who were prescribed an Trk inhibitor at our institution and 5 patients reporting withdrawal pain. Small sample size is certainly the major limitation of our report, but also reflects the rarity of *NTRK* fusions which highlights a real-world population. Another potential obstacle is that withdrawal pain was predominantly self-reported by patients, so there is a possibility of underreporting of withdrawal pain upon Trk inhibitor discontinuation.

In conclusion, this article illustrates the presence of withdrawal pain syndrome in patients stopping a Trk inhibitor treatment and highlight the need for patient education to avoid missing any doses and for development of a guideline for Trk inhibitor discontinuation.

AC researched literature, conceived the study, and gained ethical approval. AC and HK were involved in data analysis. AC wrote the first draft of the manuscript. All authors reviewed and edited the manuscript and approved the final version of the manuscript.
